# Effect of Multiple-Cycle Collections of Conditioned Media from Different Cell Sources towards Fibroblasts in In Vitro Wound Healing Model

**DOI:** 10.3390/pharmaceutics16060767

**Published:** 2024-06-05

**Authors:** Nur Izzah Md Fadilah, Mh Busra Fauzi, Manira Maarof

**Affiliations:** Centre for Tissue Engineering and Regenerative Medicine, Faculty of Medicine, Universiti Kebangsaan Malaysia, Kuala Lumpur 56000, Malaysia; izzahfadilah@ukm.edu.my (N.I.M.F.); fauzibusra@ukm.edu.my (M.B.F.)

**Keywords:** conditioned medium, dermal fibroblast, Wharton jelly, secretory protein, wound healing

## Abstract

Conditioned media refers to a collection of the used cell culture media. The goal of this study was to evaluate the possible impacts of different conditioned media collected across a number of cycles on the fibroblast proliferation, migration, and profiles of protein release. Human dermal fibroblast (HDF) cells and Wharton jelly mesenchymal stem cells (WJMSC) were cultured and incubated for 3 days prior to being harvested as cycle-1 using the serum-free media F12:DMEM and DMEM, respectively. The procedures were repeatedly carried out until the fifth cycle of conditioned media collection. An in-vitro scratch assay was conducted to measure the effectiveness of wound healing. Collagen hydrogel was combined separately with both the Wharton jelly-conditioned medium (WJCM) and the dermal fibroblast-conditioned medium (DFCM) in order to evaluate the protein release profile. The conditioned medium from many cycles had a lower level of fibroblast attachment than the control (complete medium); however, the growth rate increased from 100 to 250 h^−1^, when supplemented with a conditioned medium collected from multiple cycles. The wound scratch assay showed that fibroblast cell migration was significantly increased by repeating cycles up to cycle-5 of DFCM, reaching 98.73 ± 1.11%. This was faster than the rate of migration observed in the cycle-5 of the WJCM group, which was 27.45 ± 5.55%. Collagen hydrogel from multiple cycles of DFCM and WJCM had a similar protein release profile. These findings demonstrate the potential for employing repeated cycles of DFCM- and WJCM-released proteins with collagen hydrogel for applications in wound healing.

## 1. Introduction

Cell migration, angiogenesis, inflammation, creation of granulation tissue, remodelling of the extracellular matrix (ECM), and re-epithelialisation are all steps in the process of healing a wound [1]. Effective skin wound healing continues to be a major issue for global healthcare, as currently available skin substitutes and alternative therapeutics produce unsatisfactory results, and results vary with wound types caused by traumatic injuries, burns, and diabetes, where delayed healing and scarring are realities. Therefore, scientists and researchers are searching for safe and affordable wound treatment techniques in this regard. The secretory mediators or growth factors are necessary for either activating or suppressing the signalling pathways needed for wound healing during the healing process [2]. As a result, these mediators or growth factors could be applied as additional therapies for the treatment of wounds. The ability to heal exists in single-layer keratinocytes, single-layer fibroblasts, and bilayered skin constructions, particularly for tissue regeneration [3,4,5].

In recent years, conditioned medium-based therapy is getting more attention and has been used in the field of regenerative medicine for wound healing [6,7]. Conditioned media generally refers to the spent media that is used for culturing specific types of cells under a specific condition and time that contains proteins or small molecules secreted by the cells [8,9]. The cell-secreted proteins include growth factors, cytokines, chemokines, ECM, and small molecules including metabolites, ions, peptides, microvesicles, exosomes, etc. There is a great interest in the therapeutic potential of conditioned mediums because of their immunosuppressive properties, and ability to repair and regenerate damaged tissues. A conditioned medium normally contains a mixture of various proteins and small components that may work specifically or collectively in regulating cellular biological behaviours. Previous studies, specifically on dermal fibroblast-conditioned medium (DFCM), was produced by culturing human dermal fibroblasts (HDFs) and showed the presence of multiple proteins that enhance the expansion of keratinocytes and also accelerate wound healing in vitro and in vivo [10,11,12]. Interestingly, we previously described the discovery of different wound healing mediators in DFCM, including fibronectin, serotransferrin, serpin, and collagen, which are abundantly released by HDFs [12]. Besides fibroblasts, other cell sources such as mesenchymal stem cells (MSCs) also have the potential to secrete various ECM components (e.g., collagen, fibronectin, lumican, periostin), growth factors (e.g., basic fibroblast growth factor (bFGF), transforming growth factor beta 1 (TGF-β1), pigment epithelium-derived factor (PEDF), cytokines, and chemokines [13,14,15]. The MSC secretory proteins were also shown to enhance cellular migration, proliferation, angiogenesis, and accelerate wound healing in vitro and in vivo [16].

Furthermore, most of the studies had a focus on using cell-secreted proteins for therapeutic purposes [17,18,19]. Studies have also looked at how conditioned media affects skin regeneration and wound healing, but most of them have concentrated on conditioned media made from cell lines, MSCs, or adipose-derived stem cells rather than primary skin cells. For example, to stimulate macrophage and endothelial migration and improve cutaneous wound healing in vivo in Balb/C mice, Chen et al. showed that growth media conditioned by murine bone marrow-derived MSC contained significant quantities and high levels of released cytokines [20]. In additional research, Kim et al. demonstrated that adipose-derived MSC stimulated human dermal fibroblasts via paracrine actions, drastically reduced the size of the wound, and encouraged re-epithelialisation in an in vivo model [21]. Furthermore, Gangadaran et al. also demonstrated that three-dimensional (3D) culture-conditioned bone marrow MSC secretome accelerates wound healing in a burn injury mouse model [22]. Sun et al. reported that Wharton’s jelly-derived mesenchymal stem cells (WJMSCs) may serve as a promising candidate for the therapy of cutaneous wounds. They concluded that an MSC-conditioned medium secreted factors that promoted human umbilical vein endothelial cells (HUVEC) proliferation, regeneration of sebaceous glands, and angiogenesis. Importantly, MSC-CM promoted wound healing more than the positive control (epidermal growth factor), with no, or smaller, scar formation [23]. These studies collectively represent a novel cell-based therapeutic wound healing approach.

However, producing a large-scale, concentrated, conditioned medium requires a lot of cell expansion. This will increase production, workload, and labour costs, as well as space needed for culturing processes. Therefore, the evaluation of the multiple-cycle collections of the conditioned media using the same cultured cells will be an effective method for the production of a large scale of conditioned media with lower production costs. Thus, the main focus of this research was to produce conditioned media from cultured HDFs and WJMSCs in a multiple-cycle collections for the immediate treatment of skin loss. The collection of conditioned media required a lot of cells in order to produce large volumes of media that contained secreted proteins. Therefore, the multiple-cycle collections of conditioned media using the same cultured cells was effective for the production of large-scale, conditioned-media with lower production costs. This study also included measuring the protein concentration in different collection cycles of conditioned media and evaluated their effect on fibroblast proliferation and migration. Furthermore, the protein release profile from the different constructs of conditioned media with collagen hydrogel was also evaluated.

## 2. Materials and Methods

### 2.1. Cells Isolation and Culture

Human dermal fibroblasts (HDFs) and Wharton jelly mesenchymal stem cells (WJMSCs) were obtained from the Centre for Tissue Engineering and Regenerative Medicine (CTERM, Kuala Lumpur, Malaysia) primary cell bank. The revived cells were cultured with F12:Dulbecco’s Modified Eagle medium (F12:DMEM; Sigma-Aldrich, St. Louis, MA, USA) for HDFs and DMEM (Sigma-Aldrich) for WJMSCs. Both culture media were supplemented with 10% foetal bovine serum (FBS; Gibco, New York, NY, USA) and 1% antibiotic–antimycotic (Gibco) until confluence. The cells were seeded into 6-well culture plates (Greiner Bio-One, Monroe, NC, USA) and incubated at 37 °C in a 5% CO_2_ incubator. The culture media was replaced every 3–4 days. The 70–80% confluent cells were trypsinised using trypsin EDTA (Gibco/BRL, New York, NY, USA) and were sub-cultured in a T75 flask (Nunc, Rochester, NY, USA) using F12:DMEM and 10% FBS until passage 3 (P3).

### 2.2. Preparation and Collection of Multiple Cycles of Conditioned Medium

As the HDFs and WJMSCs at P3 have reached 80–100% confluence, the waste culture medium was removed, and the cells were rinsed twice with Dulbecco’s phosphate-buffered saline (DPBS, Sigma-Aldrich) to remove the excess medium or contaminants. Following this, a fresh F12:DMEM or DMEM medium without serum was added into the flasks containing HDFs and WJMSCs, respectively. The cells were incubated at 37 °C in a 5% CO_2_ incubator for 72 h. The conditioned medium from HDFs and WJMSCs (known as DFCM or WJCM, respectively) were collected as cycle-1 and stored at −80 °C prior to use. The remaining cells were supplemented again with a complete F12:DMEM + 10% FBS or DMEM + 10% FBS medium, respectively, for 3 days and replaced with a fresh F12:DMEM or DMEM medium without serum for collection of cycle-2. The steps were repeated until the 5th cycle of collections of conditioned medium, thus designated as cycle-5.

### 2.3. Protein Filtration and Concentration

The conditioned medium was concentrated using a 3-kDa Amicon Ultra-4 centrifugal filter (Merck Milipore, Darmstadt, Germany), which was centrifuged at 4000× *g* at 4 °C for 40 min to concentrate the proteins. The protein concentrations of DFCM and WJCM were evaluated using a bicinchoninic acid (BCA) assay (Sigma-Aldrich, USA), and the absorbance was measured with a spectrophotometer (Bio-Tek, Winooski, VT, USA) at 562 nm. The protein quantity was approximated by comparing the data to the protein standards’ values (Sigma-Aldrich, USA).

### 2.4. Evaluation of Protein Molecular Weight

The proteins in the multiple cycles of conditioned media and control media (fresh F12:DMEM or DMEM) were separated according to their molecular weight by using sodium dodecyl sulphate-polyacrylamide gel (SDS-PAGE). The proteins were loaded into a 3% Tris–glycine stacking gel and ran at 120 V for 15–20 min. The gel ran until bromophenol blue reached the bottom. The gel was then stained with Coomassie Brilliant Blue R-250 dye (Thermo Fisher Scientific, Waltham, MA, USA) and agitated for 1–2 h at room temperature. Following that, the gel was washed with 2–3 changes of de-staining solution until the bands were clearly visible.

### 2.5. Proliferation and Migration Rate Activities

#### 2.5.1. Fibroblast Proliferation

Fibroblast cells at P3 were used to evaluate the effect of multiple cycles of conditioned media on the fibroblast morphology and proliferation. Fibroblast cells were seeded at a density of 5 × 10^3^ cell/cm^2^ in 6-well plate (Greiner Bio-One, USA) with 75% of the complete medium (F12:DMEM + 10% FBS) and 25% of DFCM or WJCM from cycle-1 to cycle-5. The medium was replaced every 3–4 days. The cell images on day 0 and day 3 were captured, and the cells continued to be cultured until confluent. The 70–80% confluent fibroblasts were trypsinised using trypsin EDTA (Gibco/BRL, New York, NY, USA), and the growth rate of the cells was calculated based on the following equation:

(1) $$Growth\;rate\left(h^{-1}\right)=\frac{Ln\;(Cell\;concentration\;final/Cell\;concentration\;initial)}{Hour\;of\;culture}$$

Three technical replicates were performed for each biological replicate (*n* = 3).

#### 2.5.2. Immunocytochemistry Staining for Proliferative Cells

Fibroblast cells were fixed with 4% paraformaldehyde (Sigma-Aldrich, USA) for at least 15 min, permeabilised with 0.1% Triton X-100 solution (Sigma-Aldrich, USA) for 20 min, and blocked with 10% goat serum (Sigma-Aldrich, USA) for 1 h at 37 °C. Then, the cells were incubated with a rabbit anti-human Ki67 antibody (Abcam, Cambridge, UK) overnight at 4 °C. On the next day, the cells were incubated with goat anti-rabbit IgG Alexa Fluor 594 (red-fluorescent dye) (Invitrogen, Waltham, MA, USA) for 2 h at 37 °C. The cells then were counterstained with DAPI (Dako, Glostrup Kommune, Denmark) for 20 min at room temperature and observed using Nikon A1R confocal microscope (Nikon, Tokyo, Japan) for expression of proliferative marker, Ki67.

#### 2.5.3. Scratch Wound Healing Assays

Fibroblast cells were seeded in a 12-well plate (Greiner Bio-One, Monroe, NC, USA) and incubated at 37 °C and 5% CO_2_ until confluence. The confluent monolayer cells were then scratched with a sterile pipette tip in the middle of each well. The culture medium was removed, and the cells were rinsed with DPBS (Sigma-Aldrich, USA) and cultured in a basal medium with different cycles of DFCM or WJCM. The treatment was used by a ratio of 3:1, where 3 was equal to 75% culture medium and 1 was equal to 25% of the conditioned medium. The control group was treated by 25% of a pure medium. All scratch assays were performed in three technical replicates for each of three biological samples (*n* = 3). Live imaging was performed using Nikon A1R confocal microscope (Nikon) to capture the image at 20 min intervals, and the wound healing rate was calculated as the following equation:

(2) $$Wound\;healing\;rate\;(µm^{2}/h)=\frac{Initial\;area\;of\;the\;wound\;\left(µm^{2}\right)-Final\;area\;of\;the\;wound\;(µm^{2})}{Observation\;time\;(h)}$$

### 2.6. Fabrication of DFCM/WJCM with Collagen Hydrogel Construct

Collagen type I from an ovine tendon was prepared in-house according to the methodology published by Fauzi et al. (2016) [24] to produce collagen hydrogel. The pure collagen gel was neutralised by dropping 1 M of sodium hydroxide (NaOH) (Sigma-Aldrich, USA) into the solution until the pH reached 7.0. The collagen mixture was then centrifuged at 4000 rpm at 4 °C for 2 min to remove any air bubbles. The neutralised collagen solution was combined with different cycles of conditioned media and incubated at 37 °C to initiate gelation into the 3D construct.

### 2.7. Protein Release Profile

The construct with chosen collection cycles (cycle-1 and cycle-5) for both conditioned mediums were prepared in Transwell cell culture inserts (Greiner bio-one, Kremsmünster, Austria) and then incubated in 0.0015% collagenase type I (2 U/mL) (Whartington, London, UK) at 37 °C for 24 h, according to the protocol described by Sakamoto et al. (2016) [25]. The protein released to the collagenase was collected for the first 30 min and at a subsequent 2 h interval and measured using a BCA assay.

### 2.8. Sample Size Calculation

The equation is:

E = N − B − T(3)

where, 

N = Total number of Individual − 1;

B = Blocking component (Environmental effect) (0);

T = Treatment − 1;

E = Degree of freedom.

In this experiment,

T = 11 treatments (1 control, 5 cycles of 2 different conditioned medium);

N = 3 samples × treatment number.

Therefore, 

N = 3 × 11 = 33;

E = (N−1) − B − (T−1)

= (33−1) − 0 − (11−1)

= 22.

As E = 22, this shows that 3 samples of fibroblasts are sufficient for this study.

### 2.9. Statistical Analysis

The quantitative results are reported as the mean ± standard error of the mean (SEM). Statistical analysis was performed using GraphPad Prism 7.0 (GraphPad Software, La Jolla, CA, USA), and the results were analysed using two-way analysis of variance (ANOVA). The difference between the groups was significant if *p* < 0.05.

## 3. Results

### 3.1. Effect of DFCM and WJCM on the Cells Morphology

**Figure 1:**
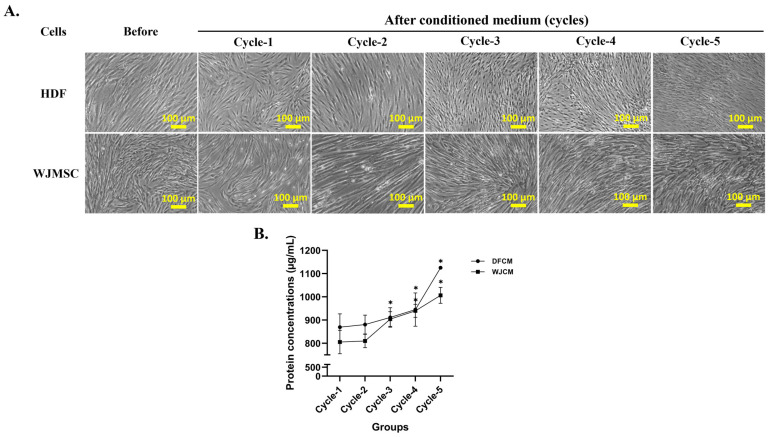
(**A**) Cell morphology during collection of multiple-cycle conditioned media, and (**B**) protein concentrations of collection multiple cycles of different cell sources (DFCM and WJCM). Data are expressed in mean ± standard deviation (SD), *N* = 3; *n* = 3. * *p* < 0.05 compared to the cycle-1 group.

Figure 1A shows the morphology of fibroblast and Wharton jelly cells after the collection of multiple cycles of the human dermal fibroblast-conditioned medium (DFCM) and Wharton jelly-conditioned medium (WJCM), respectively. The cells maintained their flat and spindle shape (elongated) throughout the collection time, up to cycle-5. The fibroblast cells did not show any significant changes during the collection of multiple cycles for DFCM and WJCM.

### 3.2. Concentrations of Secreted Proteins from DFCM and WJCM

Figure 1B shows the amount of protein concentrations through the collection of multiple cycles of both DFCM and WJCM. From the graph, both DFCM and WJCM are presented as higher protein concentrations which are above 600 µg/mL for all cycle-1 to cycle-5. This may be due to different cell sources having different capability to secrete proteins in the culture medium, since then, we guess, human dermal fibroblasts have the ability to secrete more proteins compared to Wharton jelly mesenchymal cells. This finding provides insight into our previous findings, where we demonstrated that DFCM gives higher protein concentrations [12].

### 3.3. Determination of Molecular Weight of Secreted Proteins from DFCM and WJCM by SDS-PAGE

**Figure 2:**
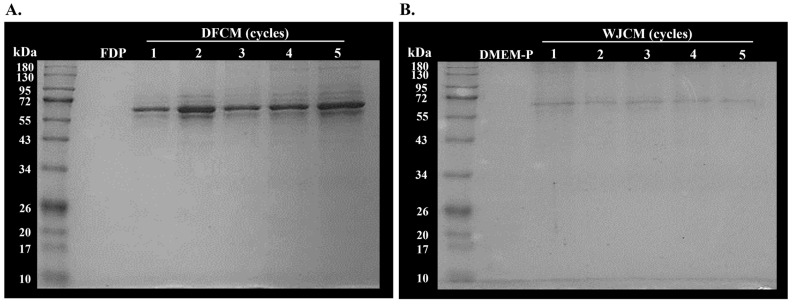
The visualisation and separate proteins of concentrated multiple cycles of (**A**) DFCM and (**B**) WJCM in comparison to the respective controls by 1D SDS-PAGE.

The secretory proteins in the multiple cycles of DFCM and WJCM were separated according to their molecular weight (MW). Figure 2A and Figure 2B demonstrated the presence of low MW proteins in the range of 55–72 kDa for all cycles for both DFCM and WJCM, respectively. Furthermore, the expression of the proteins was quite dark in the DFCM, representing that the fibroblasts had released more proteins into the culture medium. However, there are no significant differences for SDS-PAGE of secreted proteins in multiple cycles of both conditioned mediums, thus indicating that the MW of the proteins in the culture medium was maintained from cycle-1 to cycle-5.

### 3.4. Effect of DFCM and WJCM on the Fibroblast Proliferation

**Figure 3:**
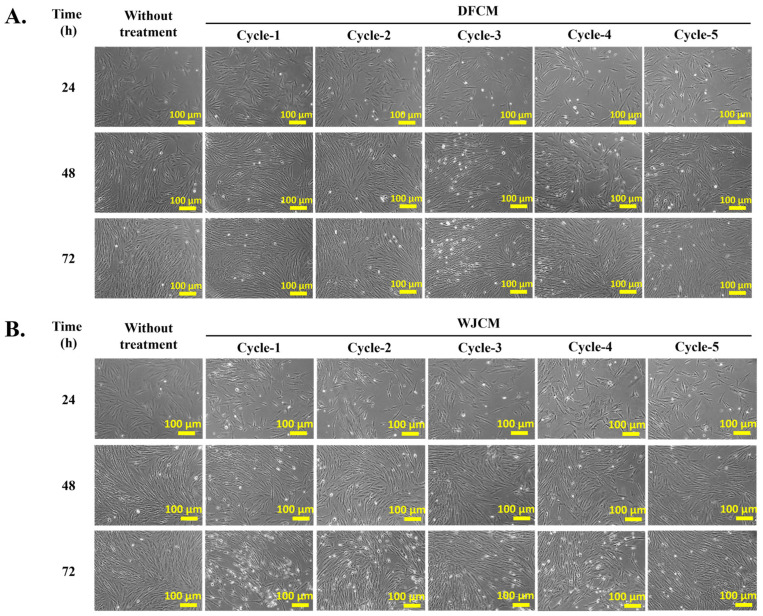
The morphology of fibroblast cells after supplementation with (**A**) DFCM and (**B**) WJCM throughout 72 h treatment.

The effect of cycle dependency for both DFCM and WJCM on fibroblast morphology, attachment, and proliferation was assessed by comparison with the negative control (complete medium). The fibroblast cells were grown well; however, there was no significant difference in the in vitro fibroblast properties between the DFCM and WJCM groups. Figure 3A and Figure 3B represent the morphology of fibroblast cells after supplementation with DFCM and WJCM, respectively.

**Figure 4:**
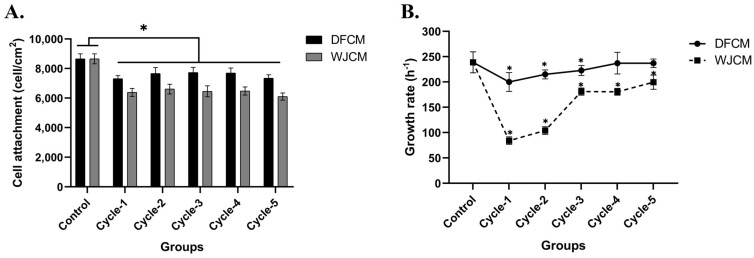
(**A**) The effect of multiple cycles of both DFCM and WJCM on fibroblast attachment. The data representation of three different experiments and expressed as mean ± SD; * *p* < 0.05 when compared with control (completed medium). (**B**) The effect of multiple cycles of both DFCM and WJCM on fibroblast growth rate. The data representation of three different experiments and expressed as mean ± SD; * *p* < 0.05 when compared with control (completed medium).

As shown in Figure 4A, the cell attachment of the DFCM and WJCM cycles resulted in similar attachment patterns that are comparable to each other. The DFCM and WJCM showed lower fibroblast attachment (DFCM= ~7000 cell/cm^2^; WJCM = ~6000 cell/cm^2^) than the control (complete medium = ~8000 cell/cm^2^); *p* < 0.05. However, the DFCM-treated groups facilitated fibroblast attachment with significantly higher efficiency than the WJCM-treated. This could be due to the higher protein concentration in the DFCM group and the same medium being used for the preparation of the DFCM and culturing of the fibroblasts as well, which was derived from F12:DMEM. According to our previous study, a fibroblast-specific medium is a medium used to culture fibroblasts and support their growth [10]. Moreover, the medium for fibroblast growth was similar to that of the skin cells and living dermis, which has high proliferative capacity [26]. Besides that, Figure 4B shows the cycle-dependent effect of DFCM and WJCM on fibroblast growth rate. As shown in the graph, the growth rate increased when the cycles for both DFCM and WJCM increased. However, the growth rate for WJCM cycles were significantly lower than the DFCM cycles (*p* < 0.05).

### 3.5. Immunocytochemistry Staining for Proliferative Cells

**Figure 5:**
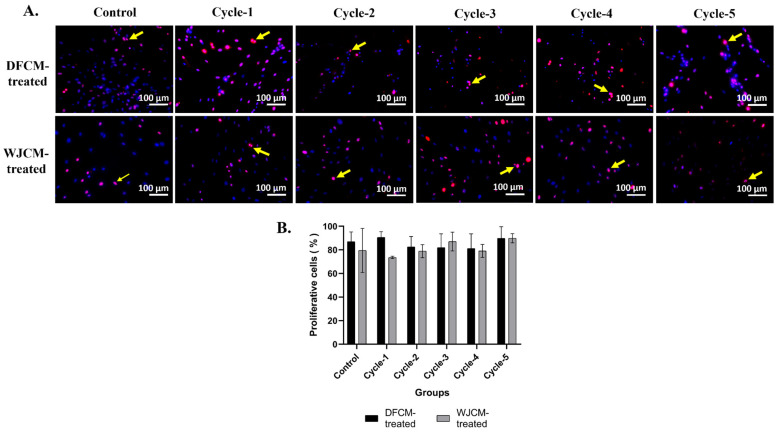
The effect of DFCM and WJCM on fibroblast proliferation. (**A**) Representative images of immunocytochemistry staining of fibroblasts supplemented by multiple cycles DFCM and WJCM, with anti-Ki67 (red) and nuclear staining (blue). The yellow arrow indicates positive expression of proliferative cells with anti-Ki67. Scale bar is 100 µm. Magnification 20×. (**B**) Quantitative evaluation (in percentage) of proliferative cells for both treated groups (DFCM and WJCM).

Immunocytochemical staining confirmed these results, where DFCM and WJCM from cycle-1 to cycle-5 had more proliferative cells and were more positively stained with Ki67, and it was comparable to the control. Figure 5A shows representative images of immunocytochemistry staining of fibroblasts treated with DFCM and WJCM and with anti-Ki67 (red) and nuclear staining (blue); meanwhile, the quantitative evaluation was presented in Figure 5B. From the results, there are observed differences in the percentages of the proliferation marker (Ki67) between the groups treated with different cycles of DFCM and WJCM. All five cycles of the DFCM-treated groups had a higher percentage of above 80% proliferative cells; meanwhile, the group treated with cycle-1 WJCM had a low percentage of proliferative cells, which was below <80%. However, these differences are not statistically significant.

### 3.6. Effect of Dermal Fibroblast-Conditioned Medium (DFCM) on Fibroblast Migration

**Figure 6:**
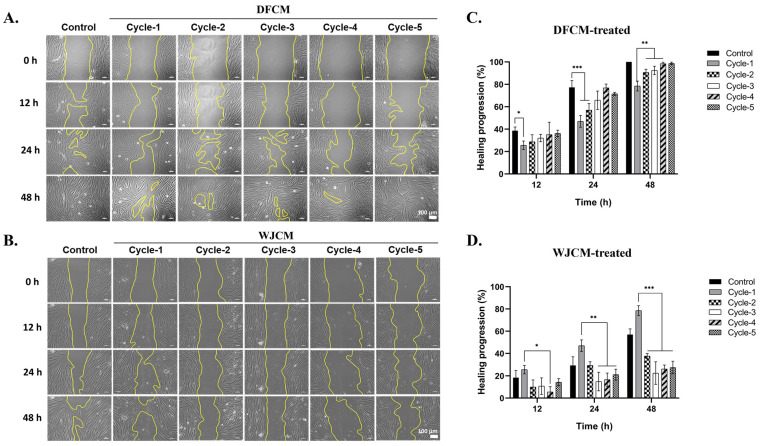
The effect of multiple cycles of (**A**) DFCM and (**B**) WJCM on the fibroblast migration for 48 h. The representative images of fibroblast migration treated with 1×–5× cycles of DFCM and WJCM. Yellow line indicates cell movement. Scale bar is 100 µm. Healing progression of multiple cycles (**C**) DFCM and (**D**) WJCM. Data were shown as mean ± SD obtained from triplicate experiments. The asterisks represent * *p* < 0.05, ** *p* < 0.01, *** *p* < 0.001.

Since cell migration plays a vital role in wound repair, a scratch assay was conducted to observe the healing progress. It is a form that mimics the wound in vitro and is evaluated in cell migration [27,28]. The cells were treated with different cycles of DFCM from cycle-1 to cycle-5. Figure 6A and Figure 6B showed the image analysis of fibroblast cell migration after two days of DFCM and WJCM treatment, respectively. In the meantime, Figure 6C and Figure 6D represented the details of the migration percentage (healing progression) of the cells. By observing the image of analysis, the multiple cycles of DFCM effectively promoted the fibroblast cell migration towards the induced gap and were compared to the untreated control. As there was treatment with more cycles of DFCM, the percentage of healing progression was observed to increase over time. After 48 h of treatment, the cycle-5 group showed 98.73 ± 1.11% healing progression compared to cycle-4 group, which was 98.49 ± 1.38%. Hence, this enhanced migration can explain the promoted cell proliferation [29]. Furthermore, DFCM is analogous to a therapeutic drug for skin wound healing since it promotes cell proliferation and migration. This is because the fundamental event in the wound repair process is cell spreading and cell migration, which permits the epithelial recovery of the denuded collagen matrix, therefore replacing missing tissue layers and cellular structures [30,31].

### 3.7. Fabrication of 3D Construct of Collagen/Conditioned-Medium and Protein Release Profile

**Figure 7:**
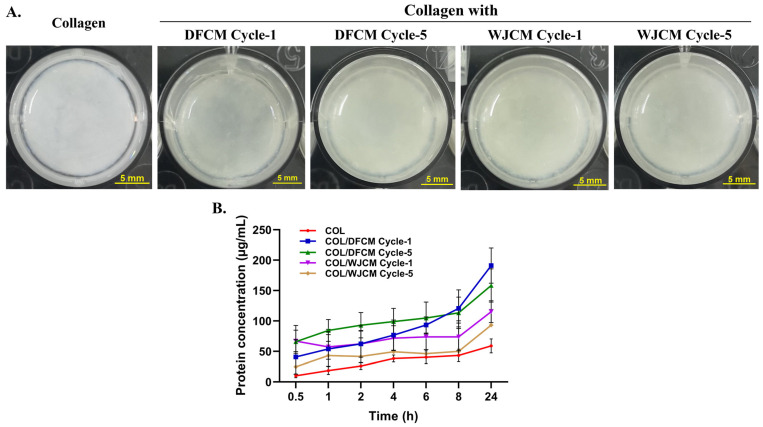
(**A**) Gross morphology and general structure of collagen hydrogel fortified with DFCM- and WJCM and (**B**) cumulative amount of protein released from collagen hydrogel over 24 h period.

The gross morphology of the 3D construct with DFCM and WJCM are shown in Figure 7A. They represented a soft, semi-solid and translucent gel-like structure. Meanwhile, Figure 7B shows the protein released from the collagen hydrogel after the 24 h experimental time. When the cumulative amount of the protein release from the collagen hydrogel were plotted against time, it was found that the protein concentrations were increased with the incubation time. After 24 h incubation, the collagen mixed with DFCM (cycle-1: 191.08 ± 2897 µg/mL; cycle-5: 158.46 ± 27.40 µg/mL) had higher protein release than the collagen mixed with WJCM (cycle-1: 115.24 ± 18.03 µg/mL; cycle-5: 93.31 ± 25.51 µg/mL) as well compared to the control (10.195 ± 3.20 µg/mL).

## 4. Discussion

The usage of skin substitutes for wound healing has been sparked by the conventional treatment of skin loss [32]. Many skin replacements have recently been employed in clinical settings to treat and heal massive and deep wound injuries [33]. Therefore, due to the increased need for skin substitutes, researchers and surgeons have teamed up to create skin substitutes or wound-care products that can aid in recovery and promote healing as well. Studies on the secretome profile, which is a representation of the proteins produced by cultured cells primarily in serum-free media, have grown recently [34,35]. The conditioned media was required and necessary for tissue repair, because it has been demonstrated to encourage host-cell proliferation and migration [36]. Therefore, research into the variables affecting dermal fibroblast and epidermal keratinocyte migration may aid in developing medicines with more precise targets for better cutaneous wound healing.

In this set of investigations, we demonstrated the different effects of two types of conditioned media, specifically DFCM and WJCM on the fibroblasts in in vitro wound healing. Over the course of the period when the conditioned medium was being collected, the morphology of fibroblasts and Wharton jelly cells was documented. Indeed, the cell’s morphology revealed no specific difference during the collection of conditioned media until five cycles. We also observed the cells that were grown in healthy conditions, even though free-serum was added. Previous studies reported that, even if not significant, there was an increase in cell numbers compared to the cells before the collection of first conditioned medium [37]. Interestingly, the fibroblasts and Wharton jelly cells can release proteins, and their conditioned media contains wound-healing mediators that may aid in promoting wound healing [36,38]. The protein concentrations increased with the multiple-cycle collections up to cycle-5. Furthermore, the secretory proteins in the DFCM and WJCM were also separated according to their molecular weight. Following that, 1D SDS-PAGE was performed on the concentrated samples of the conditioned medium. The low-molecular weight of the present proteins was in the range ~55–72 kDa for all cycles for both DFCM and WJCM. However, the DFCM showed a higher expression of these proteins, indicating that more of the fibroblasts released these proteins into the culture medium. Thus, the DFCM and WJCM may be utilised in addition to conventional medicine to treat skin injuries.

Moreover, a cell proliferation assay has been carried out to determine the changes in cell viability as a result of cell growth and division [28]. The results obtained by simulating the environment of an in vitro skin wound showed the viability, attachment, and proliferation of fibroblasts after a given amount of time to collect the conditioned medium. This finding may increase the likelihood of a better therapeutic outcome for skin wound healing. Based on the previous studies, cells secreted by growth factors or chemokines are likely targets to encourage re-epithelialisation, angiogenesis, and stimulatory activity in cutaneous wounds and, thereby, speed up the proliferation of HDFs [39,40,41]. From the attachment of cells, the DFCM-treated groups facilitated fibroblast attachment with significantly higher efficiency than the WJCM-treated groups. This is probably due to the effect of higher DFCM concentrations, which downregulated the expression of genes such as fibroblast growth factor (FGF), epidermal growth factor (EGF), platelet-derived growth factor (PDGF), and vascular endothelial growth factor (VEGF), which are crucial in cell proliferation and migration during wound healing. The homeostasis of the microenvironment and the coordination of the intricate physiological response to wounds are both crucially dependent on the fibroblast cells. Growth factors generated by inflammatory cells prompt fibroblasts to move towards a wound, multiply, and secrete a collagen-rich extracellular matrix in the early stages of wound healing [42]. These findings were validated by immunocytochemical staining, which showed that DFCM and WJCM from cycle-1 to cycle-5 had more proliferative cells and were more strongly stained with Ki67 while being comparable to the control.

By rapidly proliferating and migrating to fill the wound, fibroblasts play a positive role in wound healing. They also develop into myofibroblasts, which compress the wound, thus promoting wound repair [43]. A stimulation of fibroblast migration and proliferation results in re-epithelialisation, the creation of granulation tissue, and tissue remodelling [44]. Thus, cell migration is crucial for wound healing progression. An in vitro scratch assay was performed to track the course of the test sample’s healing towards examination of cellular migration [45]. Different factors can influence migration and, as such, proper wound healing [46]. From the results obtained, the numerous DFCM cycles successfully encouraged fibroblast cell migration to the induced gap. According to a recent study by Smith et al. [47], co-culturing murine MSC with HDFs in the presence of bovine and horse serum, with each cell type isolated in a modified Boyden chamber, increased dermal fibroblast migration and proliferation. Genes involved in interactions between cells and their environment were altered in expression because of the dermal fibroblasts’ reaction.

The major component of the ECM is collagen, where it is also the most widely employed biomaterial due to its availability, low immunogenicity, biocompatibility, biodegradability, and hydrophilicity [48]. In the present study, we fabricated a 3D construct as a hydrogel by using collagen fortified with DFCM and WJCM. The protein-release profile demonstrated that the collagen hydrogel mixed with DFCM and WJCM can sustain the protein to be released within a 24 h incubation time. However, the collagen hydrogel with multiple-cycle collection of DFCM and WJCM showed a similar protein release profile. Another study has also proven that collagen hydrogel is suitable for loading DFCM and can slowly release it to enhance wound healing. Due to the collagen hydrogel’s favourable swelling, degradation, chemical, and mechanical properties that resemble the typical biomaterials used for skin substitutes, it may be employed as an acellular skin substitute [49]. These findings highlight the possibility of using cell-secreted proteins from multiple-cycle collection of DFCM and WJCM with collagen hydrogel for wound healing.

## 5. Conclusions

The multiple-cycle collections of conditioned media could be an effective method to produce large-scale conditioned medium. It was seen as a successful or efficient approach. In the context of biological or cell cultures, efficiency could refer to obtaining a high yield of the desired substance or achieving a particular biological effect. The use of conditioned media from different cell sources resulted in lower fibroblast attachment than the control (complete medium); nevertheless, the growth rate increased when supplemented with conditioned medium collected from more cycles. The wound scratch assay confirmed that multiple cycles of conditioned media significantly increased fibroblast cell migration, which occurred faster than in the control group. These findings suggest the possibility of using cell-secreted proteins from multiple-cycle collections of DFCM and WJCM combined with collagen hydrogel to promote wound healing. The collagen hydrogel with multiple-cycle collections of DFCM and WJCM had a similar protein release profile. Therefore, this DFCM- and WJCM-fortified collagen hydrogel is a potential off-the-shelf product to be applied for immediate treatment of skin injuries. Also, it could be a readily available product that can be used to treat skin damage right away.

## Data Availability

Data are contained within the article.
